# A method to detect single-nucleotide polymorphisms accounting for a linkage signal using covariate-based affected relative pair linkage analysis

**DOI:** 10.1186/1753-6561-5-S9-S84

**Published:** 2011-11-29

**Authors:** Yeunjoo E Song, Junghyun Namkung, Robert W Shields, Daniel J Baechle, Sunah Song, Robert C Elston

**Affiliations:** 1 Department of Epidemiology and Biostatistics, Case Western Reserve University, 10900 Euclid Avenue, Cleveland, OH 44106, USA; 2 Department of Electrical Engineering and Computer Science, Case Western Reserve University, 10900 Euclid Avenue, 321 Glennan Building, Cleveland, OH 44106, USA

## Abstract

We evaluate an approach to detect single-nucleotide polymorphisms (SNPs) that account for a linkage signal with covariate-based affected relative pair linkage analysis in a conditional-logistic model framework using all 200 replicates of the Genetic Analysis Workshop 17 family data set. We begin by combining the multiple known covariate values into a single variable, a propensity score. We also use each SNP as a covariate, using an additive coding based on the number of minor alleles. We evaluate the distribution of the difference between LOD scores with the propensity score covariate only and LOD scores with the propensity score covariate and a SNP covariate. The inclusion of causal SNPs in causal genes increases LOD scores more than the inclusion of noncausal SNPs either within causal genes or outside causal genes. We compare the results from this method to results from a family-based association analysis and conclude that it is possible to identify SNPs that account for the linkage signals from genes using a SNP-covariate-based affected relative pair linkage approach.

## Background

Owing to the complexity of the genetic models underlying complex traits, model-free linkage methods, which do not require the specification of a disease model, are a popular choice. With these methods, inclusion of covariates increases the power to detect linkage [1], provided that the covariates reflect underlying locus heterogeneity. The method allows the genetic relative risk to depend on the covariate so that, in effect, the allele sharing at the marker locus differs for different values of the covariate. A general conditional-logistic model developed by Olson [2] provides a unified framework to incorporate covariates, and this model is implemented in LODPAL (SAGE, version 6.1.0) [3]. A modified one-parameter model has been proposed [4], so that only one additional parameter per covariate is required.

To identify single-nucleotide polymorphisms (SNPs) that may explain the observed linkage signals, several researchers have developed methods for an affected pair analysis [5-10] and for quantitative trait linkage analysis [11]. Among these studies, Houwing-Duistermaat et al. [8] proposed using Olson’s conditional-logistic model with a genotype-based covariate to explain the linkage signals. They applied the method to three SNPs and five markers in the Genetic Analysis Workshop 14 data, and they confirmed a SNP that explained a linkage peak. However, the statistical properties of this method still need to be studied. The large numbers of SNPs from exome sequencing data, along with the identical-by-descent (IBD) allele sharing from fully informative markers in the Genetic Analysis Workshop 17 (GAW17) data set [12], provide a good opportunity to evaluate this approach; hence our purpose here is to evaluate this new method in depth.

## Methods

### Phenotype data

We analyzed all 200 replicates of the GAW17 family data set. The binary affected status was analyzed as the main trait of interest, and the affected relative pairs from all eight extended pedigrees were used.

Based on the knowledge of the underlying simulating model, we included Age, Sex, and Smoking status as covariates in all analyses. Using the method of Doan et al. [13], we combined these three covariates into a single variable, a propensity score (PS), as a means of allowing for multiple covariates with the addition of only 1 degree of freedom (df). In each replicate, the PS values were estimated by taking the predicted probability of being affected, given the set of covariates, after fitting a logistic regression of affection status on the given covariates with all 698 individuals using R, version 2.10.1 [14].

### Marker data

We used the IBD sharing values for the 3,205 genes from 22 autosomal chromosomes. After removing the SNPs with no variability in the data set or with no LOD score result from LODPAL, the average number of SNPs remaining for the analysis in each replicate was 9,069 out of 24,487 total SNPs. Among these 9,069 SNPs, 8,912 were in noncausal genes, 126 were noncausal SNPs in causal genes, and 31 were causal SNPs in causal genes. Each gene contained 1 to 231 SNPs. Using an additive coding based on the number of minor alleles, we recoded each SNP as 0, 1, or 2.

### Analysis

In the general conditional-logistic model by Olson [2], the likelihood ratio (LR) for a relative pair *r* is written:(1)

where *λ_i_* is the relative risk of disease for an individual who shares *i* alleles IBD with an affected relative, *f_ri_* is the prior probability that a pair will share *i* alleles IBD, and  is the estimated probability that a pair will share *i* alleles IBD conditional on available marker data. The model is parameterized in terms of the logarithms of relative risk, so:(2)

where the *δ_ij_* are the parameters associated with the covariate *x_j_*, with *β*_0_ and *δ*_0_ = 0. We use a one-parameter model so that only one additional parameter is estimated for each included covariate. The asymptotic distribution of the LR statistics (i.e., 4.605 × LOD score) for the one-parameter model is a 50:50 mixture of a chi-square distribution with *K* df and a chi-square distribution with *K* + 1 df when there are *K* covariates in the model and the relative pairs are independent.

The first model in our analysis includes one covariate PS, so:(3)

The second model included two covariates, PS and a SNP, incorporating the SNPs in a gene one by one as an additional covariate, so:(4)

We evaluate the LOD score increases from the first model to the second model (LodDiff) to detect SNPs that differentially account for the linkage signals.

In each replicate, the LodDiff values are calculated for all available SNPs. Then, the mean LodDiff values are calculated for three different groups of SNPs: SNPs in noncausal genes, noncausal SNPs in causal genes, and causal SNPs in causal genes. The distributions of these mean LodDiff values over 200 replicates are compared. Again in each replicate, all SNPs are sorted and divided into 10 equal partitions (deciles) according to their LodDiff values, and the proportion of true causal SNPs within each partition is checked. We report the mean proportion values over 200 replicates.

To conduct family-based association analysis using the residuals obtained from the logistic regression model with Age, Sex, and Smoking as covariates, we use the ASSOC program in SAGE (version 6.1.0). ASSOC performs a likelihood-based regression unconditional on parental genotype. The analysis model includes a SNP as a fixed effect and a polygenic component as a random effect. To account for the nonnormal distribution of the residuals, we apply the George-Elston transformation. The –log(*p*-value) is summarized in the same way as the LodDiff value was from 200 replicates.

## Results and discussion

**Figure 1:**
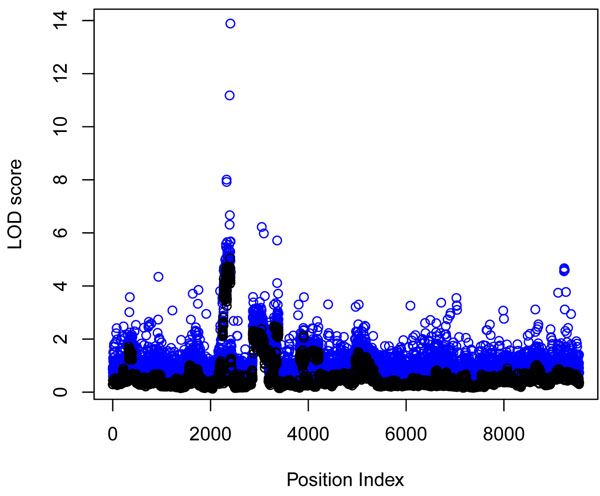
**LOD scores from the model without SNP covariate and the model with SNP covariate.** LOD score is plotted against SNP location. The first model without a SNP covariate is plotted in black, and the second model with a SNP covariate is plotted in blue.

**Figure 2:**
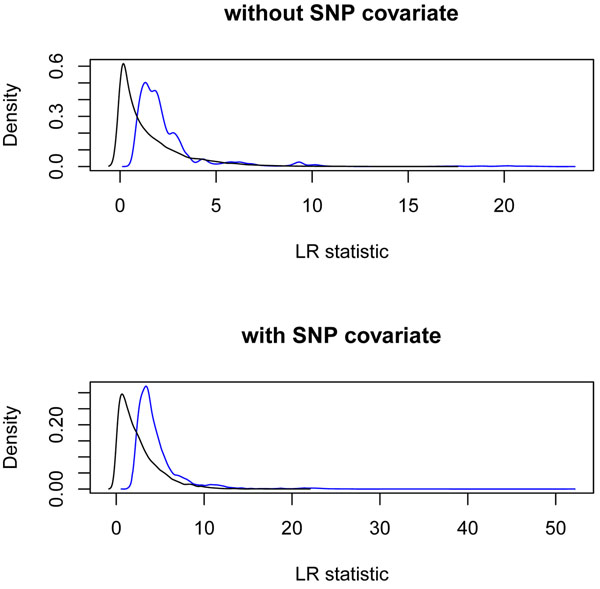
**Observed LR statistics and theoretical mixture distribution**. The density of observed LR statistics from LOD scores using SNPs in noncausal genes is plotted. For the first model without a SNP covariate, the theoretical distribution is a 50:50 mixture of a chi-square distribution with 1 df and a chi-square distribution with 2 df because we have one covariate. For the second model with a SNP covariate, the theoretical distribution is a 50:50 mixture of a chi-square distribution with 2 df and a chi-square distribution with 3 df because we have two covariates. The blue curve is for the observed LR statistics; the black curve is for the theoretical values.

In Figure 1 we plot the mean LOD scores from 200 replicates for each SNP for both models. In the first model without a SNP covariate, several genes in chromosomes 4 and 6 were significant (LOD score > 3.0), and the inclusion of an additional SNP covariate increased the LOD score substantially in the second model. In Figure 2 the density plots of observed LR statistics from these mean LOD scores using SNPs in noncausal genes are compared with the theoretical 50:50 mixture of a chi-square distribution with 1 df and a chi-square distribution with 2 df for the first model and with a 50:50 mixture of a chi-square distribution with 2 df and a chi-square distribution with 3 df for the second model.

**Figure 3:**
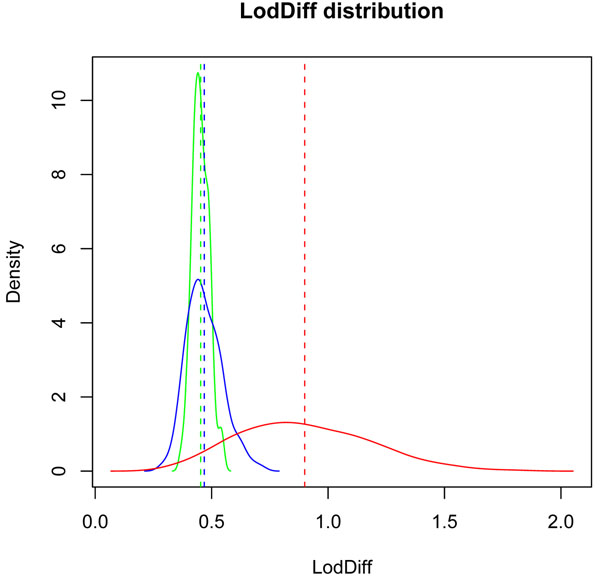
**Density plots of LodDiff values.** The distributions of LodDiff values for three groups of SNPs: noncausal SNPs in noncausal genes (green), noncausal SNPs in causal genes (blue), and causal SNPs in causal genes (red). The dashed line in the same color as the curve indicates the mean location for each distribution.

The density plots of the mean LodDiff values for the three groups of SNPs are shown in Figure 3. From the distribution of LodDiff, we found that the inclusion of causal SNPs in causal genes increased the LOD scores more than the inclusion of noncausal SNPs did either within causal genes or outside causal genes. The overall mean LodDiff values were 0.45 (± 0.04) for noncausal SNPs in noncausal genes, 0.47 (± 0.07) for noncausal SNPs in causal genes, and 0.89 (± 0.3) for causal SNPs in causal genes.

**Figure 4:**
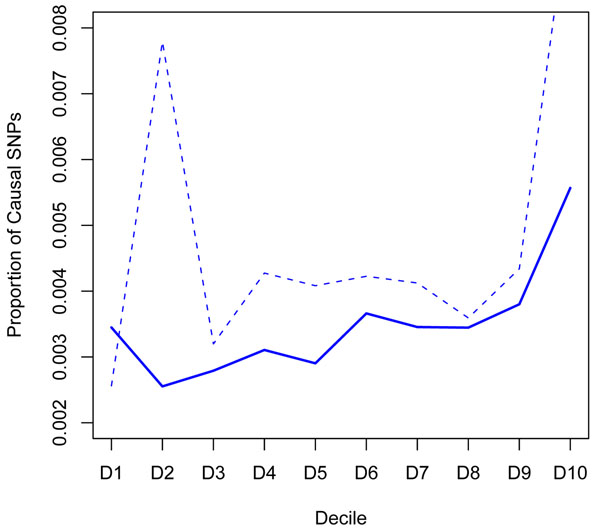
**Proportions of causal SNPs in the LodDiff distribution.** The plot shows the proportions of the causal SNPs in each decile of the sorted LodDiff values from the linkage analysis (solid line) and of the sorted –log(*p*-value) from the family-based association analysis (dashed line).

The plot of the proportions of the causal SNPs in each decile of the sorted mean LodDiff values, starting with the bottom decile, is shown in Figure 4. A clear tendency can be seen for this proportion to increase, implying that this approach may be able to detect causal SNPs. The proportions of causal SNPs in the ten deciles of –log(*p*-values) from the family-based association analysis is presented with a dashed line for comparison.

From the ordered mean LodDiff values of SNPs, five causal SNPs (C4S4935, C6S2981, C10S3109, C4S1878, and C8S442) are included in the top 5% (Table 1). Among these five causal SNPs, the top three SNPs (C4S4935, C6S2981, and C10S3109) are included in the top 1%, and two of these SNPs are in the top 0.01%. From the family-based association analysis, six causal SNPs (C10S3109, C6S2981, C4S4935, C4S1878, C9S444, and C13S523) are included in the top 5% of significant SNPs, four of which are among the SNPs identified by the covariate-based linkage analysis.

**Table 1:** Causal SNPs within the top 5% of SNPs

Chromosome	SNP	LodDiff	Gene	Minor allele frequency	Effect	*p*-value
4	C4S4935	9.09	*VEGFC*	0.000717	1.35726	0.0000813
6	C6S2981	4.01	*VEGFA*	0.002152	1.20645	0.0000201
10	C10S3109	2.20	*SIRT1*	0.000717	0.51421	0.0000112
4	C4S1878	1.45	*KDR*	0.164993	0.13573	0.0022138
8	C8S442	1.12	*LPL*	0.015782	0.49459	NA

The information on five causal SNPs within the top 5% of SNPs from the ordered LodDiff values are shown. The *p*-values in the last column are from the family-based association analysis.

In addition, we checked the correlations between LodDiff and other properties of SNPs. The correlation between the LodDiff values and the number of additional SNPs in the gene being considered was 0.05. The correlation with the minor allele frequency of the SNP included was 0.07 and 0.19 with the effect size. Our analysis did not consider the linkage disequilibrium structure. Linkage disequilibrium between SNPs within a gene and SNPs in different genes might affect the effectiveness of LodDiff. Further work is needed to investigate this matter.

## Conclusions

We investigated the possibility of identifying SNPs that account for the linkage signals coming from genes using a covariate-based affected relative pair linkage approach. Further research is needed to study the statistical properties and the empirical null distribution to evaluate the significance of any result.

## Competing interests

The authors declare that there are no competing interests.

## Authors’ contributions

YES conceived and designed the study, performed the analyses, and drafted the manuscript. JN carried out association analyses and helped draft and revise the manuscript. RWS helped with data preparation and edited the manuscript. DJB edited the manuscript and helped revise the manuscript. SS helped with visualization of the results. RCE participated in the design of the study and revised the manuscript. All authors read and approved the final manuscript.
